# On the Means to Be Employed in Syncope from Hemorrhage after Surgical Operations

**Published:** 1861-01

**Authors:** 


On the Means to be Employed in Syncope from Hemorrhage after Surgical Operations.—M. Debout, editor of the *Bulletin de Thérapeutique*, has published in a late number of that periodical, a valuable article wherein he shows by facts and arguments, that this syncope is not to be treated like the fainting which occurs after parturient hemorrhage. M. Debout concludes with the following rules:

1. Syncope following traumatic hemorrhage is a more serious symptom than the fainting resulting from uterine hemorrhage, both on account of the nature of the blood lost and its less rapid escape; it may, therefore, be affirmed, that the means found successful in the latter complication are not, as a consequence, likely to be efficient in the former.

2. As syncope, in traumatic hemorrhage, is the result of want of stimulus to the nervous centers, the remedy will consist in putting the patient upon his back, and in lessening the extent of the circulation by abdominal pressure.

3. As we are to be especially anxious for the energy and duration of our therapeutic interference, we should add to the physical action of the means above alluded to the stimulation of the heated steel knob rapidly carried over various parts of the cutaneous surface, and assist this operation by enemata of wine. This syncope is so perilous that all the above measures combined will never be too powerful.

M. Debout thinks that transfusion of blood, in these cases, is not to be relied upon.—*London Lancet.*

